# Association of interleukin-8 polymorphism (+ 781 C/T) with the risk of oral Lichen Planus disease

**DOI:** 10.1186/s12903-023-03088-7

**Published:** 2023-06-20

**Authors:** Haniyeh Ghasemi, Hamid Reza Mozaffari, Maryam Kohsari, Masoud Hatami, Kheirollah Yari, Mohammad Hesam Marabi

**Affiliations:** 1 Student Research Committee, School of Dentistry, Kermanshah University of Medical Sciences, Kermanshah, Iran; 2 Medical Biology Research Center, Health Technology Institute, Kermanshah University of Medical Sciences, Kermanshah, Iran; 3 Department of Oral and Maxillofacial Medicine, School of Dentistry, Kermanshah University of Medical Sciences, Kermanshah, Iran; 4 Department of Clinical Biochemistry, Kermanshah University of Medical Sciences, Kermanshah, Iran

**Keywords:** Oral Lichen Planus, IL-8, Genetic polymorphism, PCR-RFLP, SNP

## Abstract

**Background:**

Oral Lichen Planus (OLP) is a chronic inflammatory mucosal disease. The pathogenesis of OLP is unknown. The Single Nucleotide Polymorphism (SNP) that occurs in the regulatory position + 781 could affect the expression of interleukin-8. This polymorphism is probably associated with increased serum levels of IL-8. The current study aimed to investigate the genotype and allele frequencies of IL-8( + 781 C/T) in OLP patients and whether it is associated with the severity of OLP disease in an Iranian population.

**Methods:**

Three milliliters of saliva were taken from 100 patients with OLP and 100 healthy individuals who were matched in age and gender. After DNA extraction from saliva samples of patients and healthy individuals, the genotype of IL-8 at position + 781 is detected using the PCR-RFLP method. The results were analyzed using SPSS software.

**Results:**

Frequency of C/C, T/C, and T/T genotypes at position IL-8 + 781 gene in the patient group were 47%, 41%, and 12%, respectively, and in the control group, were 37%, 42%, and 21%. The difference between the two groups regarding allele frequency distribution was statistically significant (χ^2^ = 3.86, p = 0.049, 95% CI = 0.44-1, OR = 0.66). Our results indicated the significantly higher frequency of the TT genotype in the erosive OLP compared to the nonerosive group (p = 0.03, OR = 0.89, 95% CI = 0.49–1.6).

**Conclusion:**

This study depicted the difference in the frequency of SNP IL-8 + 781 C/T allele in the patient and control groups had a significant association with the risk of OLP. In addition, our data revealed that IL-8 + 781 C/T polymorphisms might be associated with the severity of OLP in the Iranian population.

## Background

Oral Lichen Planus (OLP) is currently regarded as one of the potentially malignant diseases of the oral mucosa. OLP is a chronic and recurrent inflammatory autoimmune disorder [1, 2]. Based on WHO, the criteria for OLP diagnosis include the existence of relatively symmetrical bilateral white reticular lesions that sometimes could be accompanied by erosive and/or atrophic lesions [1, 3]. A meta-analysis in 2020 showed approximately 1.01% global prevalence for OLP that South America (1.74%) and North America (0.43%) had higher and lower prevalence, respectively [2]. OLP mainly affects the buccal mucosa, tongue, and gums and appears in other places. The lesions represent bilaterally and frequently occur as a combination of clinical subtypes [4]. The occurrence of OLP is more than the cutaneous form and is a treatment-resistant form of the disease [5]. Adults aged 30 to 70 are more prone to OLP, especially affecting women [6]. However, OLP rarely occurs in children, and most of the time, patients are unaware of their disease [7]. According to the clinical manifestation, OLP is divided into different types such as reticular, erosive, popular, atrophic, plaque-like, and bullous [8]. The reticular and erosive forms are the considerably common type of OLP [9]. The investigations showed OLP increased the risk of oral squamous cell carcinoma. Therefore OLP is considered a potentially malignant disorder [10, 11]. Between various types of OLP, the atrophic and erosive forms and plaque-like lesions on the back of the tongue have more malignant potential [7]. Several studies indicate multiple factors such as dental materials, stress, genetics, liver diseases like Hepatitis C virus, smoking, and alcohol consumption can contribute to OLP [7, 12, 13]. The etiology of OLP is undefined, but the hypotheses suggest that the immune system, specifically T cells, strongly correlates with OLP [14].

Cytokines play a crucial role in OLP pathogenesis [15]. Interleukin-8 (IL-8) is correlated with changes in the vital immune processes, for instance, chemotaxis and NETosis [16]. In healthy tissues, the interleukin-8 concentration of tissues is minor. However, in response to pro-inflammatory cytokines, tumor necrosis factor-alpha (TNF-α) and interleukin-1, and bacterial infection, it rapidly increases from 10 to 100 fold base value [17]. Interleukin-1 and TNF-a stimulate Keratinocytes, macrophages, T cells, endothelial cells, and fibroblasts which can cause the release of a considerable amount of IL-8 and raises the penetration of cytotoxic T cell in OLP lesions [11]. The gene polymorphisms of interleukin-8 are associated with susceptibility to several diseases such as Behçet’s, multiple sclerosis, osteoarthritis, systemic lupus erythematosus nephritis, osteosarcoma, periodontitis, oral and different cancer types [18–20]. Previous studies have revealed that interleukin-8 gene polymorphisms at positions − 251 and + 781 impacted interleukin-8 expression [21]. Recently, the association of different polymorphisms of the IL-8 gene and other cytokines with the occurrence and severity of OLP has been reported [22–25]. Therefore, the current study was designed to investigate the association of IL-8 + 781 SNP with the risk and severity of OLP disease in an Iranian population.

## Methods

### Study group and sampling

A sum of two hundred individuals, including 100 OLP patients (65 erosive and 35 nonerosive subtypes, 29 males and 71 females, mean 48 years) and 100 healthy volunteers (28 males and 72 females, mean 49 years) were included in our study. Our study groups matched in gender and age. An oral specialist doctor diagnosed OLP patients according to the clinical manifestations, pathological results, and review of the patient’s history. Patients were recruited from Kermanshah University of Medical Sciences clinics and outpatient clinics. Inclusion criteria for OLP patients were as follows: 1; diagnosis according to the definition of OLP by the World Health Organization [3], 2; did not receive any relevant treatment before inclusion in this study. Exclusion criteria were accompanying any malignancy or any other inflammatory/autoimmune diseases and systemic disorders such as diabetes, cardiovascular disease, hemorrhagic disorders, and chronic hepatitis C. Also, we excluded pregnant women, patients with suspected oral lichenoid lesions, signs of dysplasia, patients that received systemic or topical steroids over the past three months, and patients with a history of smoking or consumption of alcohol from the study. The demographic and clinical features of OLP and control groups are shown in Table 1.

**Table 1:** Clinical and demographic characteristics of OLP and control individuals

Variable	OLP (N = 100)	Control (N = 100)
**Age (years)**		
< 18 19–30 31–45 46–65 > 66	0 9 36 48 7	1 5 31 58 5
Mean	48	49
**Sex**		
Male	29	28
Female	71	72
**Clinical classification**		
Erosive:	65	-
Non-erosive:	35	-

The sample size was defined based on the genotype frequency of IL-8 + 781 [22] using the following formula:

$$n = \frac{{{{({z_1} - \alpha /2 + {z_1} - \beta )}^2}({p_1}{q_1} + {p_2}{q_2})}}{{{{({P_1} - {P_2})}^2}}}$$

A sample size of ~ 93 was calculated for each case and control group (the alpha value of 0.05 and beta value of 20%), but to ensure sample adequacy, 100 individuals were studied in each group.

Unstimulated saliva samples were collected from each participant with the spitting method [26]. Participants were told not to smoke, use mouthwash, eat, drink, and brush their teeth for at least 2 h before taking a saliva sample. Then, the subjects were instructed to spit (without stimulation) three ml of saliva in a falcon tube (15 ml). Saliva samples were transferred to the laboratory with ice, and samples were centrifuged at 10,000 g for 5 min and the obtained supernatant was stored at -20^o^C until use.

The purpose of the research was explained to all participants, and after confirming and signing the consent form, they entered into the investigation. The study design and protocol were approved by the Ethics Committee of Kermanshah University of Medical Sciences )IR.KUMS.REC.1400.423), and written informed consent for participation was obtained from the informed consent has been obtained from all participants and their legal guardians. All methods were carried out in accordance with relevant guidelines and regulations in the Declaration of Helsinki.

### Genetic analysis

**Fig. 1:**
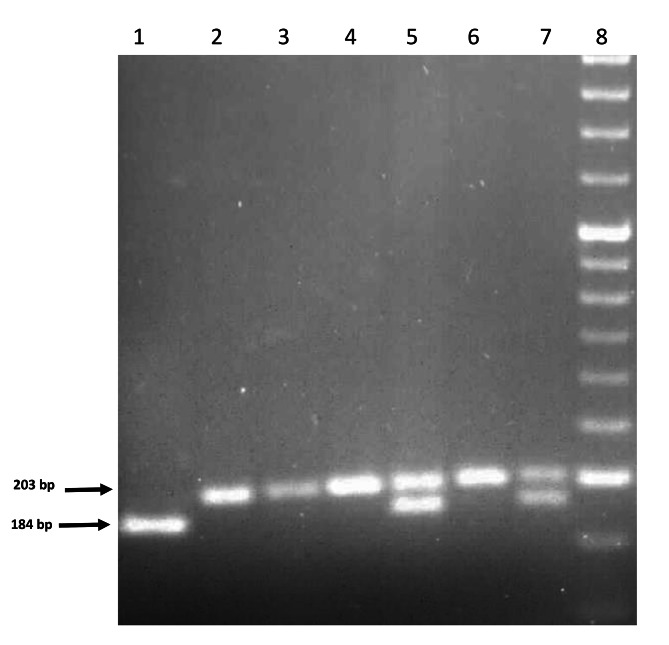
Electrophoresis pattern of PCR-RFLP products for position + 781 of the IL-8 gene. Lanes 2, 3, 4, and 6 are samples with T/T genotype; Lane 1 is with the C/C genotype, Lanes 5 and 7: are samples with the T/C genotype. Lane M: DNA size marker (50 bp)

Extraction of DNA was carried out using the standard kit (DNrich saliva kit, Cat. No. AESDX1010) for extracting DNA from the saliva of Azma Elixir Pajooh Company according to its protocol. PCR-restriction fragment length polymorphism (PCR-RFLP) method is used for genotyping. PCR amplified IL-8 gene with a set of primers, 5′-CTC TAA CTC TTT ATA TAG GAA TT-3′ (forward) and 5′-GAT TGA TTT TAT CAA CAG GCA-3′ (reverse) [27]. PCR amplifications were carried out with 500 ng of DNA as template stand, PCR buffer (1X), 12.5 ml of 2X PCR master mix (Sinacolon, Tehran, Iran), 0.5 µM of each primer in a final volume of 25 µl. The PCR parameters were: initial denaturation step at 95 °C for 7 min, followed by 30 cycles of denaturation step at 95 °C for 45 s, annealing step at 50 °C for 60 s, extension step at 72 °C for 45 s, and a final extension step at 72 °C for 10 min. After PCR, 10 µl of amplified products (with 203 bp) were digested with five units of *Eco*RI enzyme at 37 °C for 15 h. The digested products were electrophoresed on a 2% agarose gel stained with Gel Red under ultraviolet light. The band patterns were 203 bp for TT homozygotes; 203, 184, and 19 bp for CT heterozygotes; 184 and 19 bp for CC homozygotes (Fig. 1) [28].

### Statistical analysis

The significant differences in frequency distributions of alleles and genotypes between control and patient groups were analyzed by SPSS (Version 16) with Pearson’s chi-square assay. The logistic regression model was used to calculate the 95% confidence interval and Odds ratios (OR). Data on quantitative characteristics are expressed as means ± standard deviations. Results were considered statistically significant when p < 0.05.

## Results

Demographic and clinical features of OLP patients and control individuals are demonstrated in Table 1. Our result indicated that patients and controls were matched for age and sex. The highest number of patients was in the age range of 45–65 years. As indicated in Table 1, approximately 70% of individuals in both groups were women. Among the individuals in the OLP group, 65% of them were clinical erosive and 35% were classified as nonerosive subtype. The electrophoresis pattern of some of the PCR-RFLP products of the IL-8 + 781 SNP is demonstrated in Fig. 1. Distribution of IL-8 + 781 SNP genotypes in both OLP and control groups did not deviate from the Hardy-Weinberg equilibrium (χ^2^ = 0.42, χ^2^ = 1.9, respectively, p > 0.1).

The frequency of genotypes and alleles of studied individuals is shown in Table 2. The frequency of CC, TC, and TT genotypes at position + 781 of the IL-8 gene in the patient group was 47%, 41%, and 12%, respectively, and in the control group, they were 37%, 42%, and 21%, respectively. Statistical analysis showed that the difference between the two groups in terms of allele frequency distribution was statistically significant. (χ^2^ = 3.86, p = 0.049, 95% CI = 0.44-1, OR = 0.66). As indicated in Table 2, genotype frequencies in genetic models (co-dominant, over-dominant, dominant, and recessive) were not statistically different between the case and control groups (p > 0.05).

**Table 2:** Frequency distribution of SNP IL-8 + 781 genotypes and related alleles in OLP patients and control group

Genotype IL-8 + 781		Patient group n = 100	Control group n = 100	χ2	p value
co - dominant	CC	47	37	3.65	0.16
	CT	41	42		
	TT	12	21		
dominant	CC	47	37	2.05	0.15
	CT + TT	53	63		
recessive	TT	12	21	2.94	0.86
	CC + CT	88	79		
over-dominant	CT	41	42	0.02	0.88
	TT + CC	59	58		
Allele frequency	T C	65 135	84 116	3.86	0.049

Examining the effect of this polymorphism on OLP severity indicated that the IL-8 + 781 TT genotype frequency was significantly higher in the erosive OLP group than in the non-erosive group (p = 0.03, OR = 0.89, 95% CI = 0.49–1.6). However, no significant difference was observed between IL-8 + 781 allele frequency with OLP subtypes as shown in Table 3 (p = 0.69).

**Table 3:** Frequencies of IL-8 + 781 polymorphism in erosive and non-erosive subtypes

IL8 + 781	Erosive group n = 65	Non-erosive group n = 35	χ2	p value	OR
Genotypes			11.87	0.03	0.89 (95% CI = 0.49–1.6)
Wild (CC) Hetro (CT) Mutant (TT)	35	12			
	19	22			
	11	1			
Allele					
C T	89 41	46 24	0.15	0.69	

## Discussion

The current study detected a significant association between the difference in the frequency of SNP IL-8 + 781 C/T allele in the OLP patients and controls. In addition, our data revealed that IL-8 + 781 C/T polymorphisms might be associated with the severity of OLP in the Iranian population. In our study, saliva samples were used for DNA extraction and SNP genotyping of IL-8 + 781 . Saliva has more potential advantages than other biological samples, such as easy access, non-invasive, lower overall cost and lower infection risk, and patient convenience [29]. Zhang et al. showed that levels of IL-8 and other pro-inflammatory cytokines were higher in the saliva and serum of OLP patients than in healthy subjects. They suggested that saliva could be an efficient sample for testing pro-inflammatory cytokines in OLP patients [30]. Further, Sun et al., revealed that serum IL-8 might be a more sensitive marker than IL-6 in monitoring the disease activity of OLP [11].

Cytokines play an essential role in OLP pathogenesis and progression; main polymorphisms in INF-γ, TNF-α, TNF-β, IL-4, and IL-10 genes are associated with susceptibility to OLP [31, 32]. The important polymorphisms (-251 A/T, + 396 G/T, -845 T/C, and + 781 C/T) in the regulatory regions of the IL-8 gene could affect gene transcription and protein production of IL-8 and, therefore, can contribute to increasing the susceptibility of disease [18, 33]. Sajadi et al. reported a significant association between IL-8 − 845 (T/C) polymorphism and periodontitis in the studied Iranian population [34]. Also, Houshmand et al., evaluating IL-8 SNPs in Iranian people, concluded that IL-8 gene polymorphism might be protective against periodontitis [35].

Previous studies revealed that the OLP erosive subtype has severe symptoms and clinical manifestations; therefore, it is considered a severe subtype compared to the non-erosive subgroup [36]. Therefore, we analyzed the IL-8 + 781 genotype and allele frequencies between erosive and non-erosive patients. Our results indicated that the IL-8 + 781 TT genotype frequency was significantly higher in the erosive OLP group than in the non-erosive group. Therefore, this polymorphism may be associated with OLP severity.

Consistent with our results, Dan and colleagues (2010) investigated four SNPs of the IL-8 gene in 109 OLP patients and 101 healthy individuals in the Chinese population. The results revealed that the − 251 A + 781 C haplotype frequency was lower in the OLP group than in the control group (P = 0.029), while the − 251 T/+781 C haplotype frequency was higher in the OLP patients than in the healthy controls (P = 0.028). Thus, they concluded the relationship between the IL-8 polymorphisms and the severity of OLP [37].

Association of other cytokines with OLP disease evaluated in different populations; for example, Carrozzo et al. analyzed whether polymorphisms of cytokines may affect the susceptibility to OLP with the study of 13 cytokine genes with 22 single nucleotide polymorphisms. Accordingly, their results suggested that the genetic polymorphism of the first intron of the INF-c promoter gene might be an important risk factor for developing OLP lesions. At the same time, the increased frequency of the TNF-a-308 allele might contribute to skin involvement [38]. Also, the results of a meta-analysis study conducted by Shi et al. in 2017 indicated that no evidence was found to support an association between susceptibility of IL6-174G/C, IL10-819 C/T, and IL10-1082G/A with OLP disease in any genetic model. However, the significant association of IL10-592 C/A SNP was reported in all comparison models. Also, this meta-analysis could not result in a significant association between IL6-174G/C, IL-10-819 C/T, and IL-10-1082G/A and OLP susceptibility [39]. Also, a meta-analysis conducted by Zhou et al. suggests that − 308 G/A polymorphism in TNFα is a potential genetic marker for OLP [40]. Al-Mohaya et al. resulted that TNF-α (-308G/A), TNF-β (+ 252 A/G) and IL-10 (-1082G/A, -819 C/T, and − 592 C/A) polymorphisms are correlated with the risk of OLP in Saudi population [41].

To our knowledge, this is the first study to demonstrate the association of IL-8 + 781 polymorphism with the severity of OLP disease among the Iranian population. However, there were some limitations in the current study as follows: [1]; we did not study other polymorphisms and haplotypes of IL-8, [2]; we did not measure IL-8 expression and restricted to genetic analysis [3]; this result may not be generalizable to other ethnic groups, because we studied only one ethnic group.

## Conclusion

The results of the present study demonstrated the difference in the frequency of SNP IL-8 + 781 allele in the patient and control groups had a significant association with the risk of OLP. Also, our results concluded that IL-8 + 781 C/T polymorphism might be associated with the severity of OLP in the Iranian population. Therefore, our results support the genetic basis of OLP disease.

## Data Availability

All data generated or analyzed during this study are included in this published article.

## Abbreviations

OLP: Oral lichen planus
TNF-α: Tumor necrosis factor-alpha
IL: Interleukin
PCR-RFLP: PCR-restriction fragment length polymorphism
SNP: The Single nucleotide polymorphism
OR: Odds ratios
